# Focal reactive nodular gliosis: an extremely rare retinal astrocytic tumor

**DOI:** 10.3205/oc000230

**Published:** 2023-12-12

**Authors:** Saúl Villoria-Díaz, María Antonia Saornil-Álvarez, Ciro García-Álvarez, Elena García-Lagarto, Irene Bermúdez-Castellanos

**Affiliations:** 1 University Care Complex of Palencia, Department of Ophthalmology, Palencia, Spain; 2 University Clinical Hospital of Valladolid, Department of Ophthalmology, Valladolid, Spain; 3 University Clinical Hospital of Valladolid, Department of Pathology, Valladolid, Spain; 4 Río Hortega University Hospital, Department of Ophthalmology, Valladolid, Spain

**Keywords:** astrocytes, glial fibrillary astrocytic protein, gliosis, Ki-67 antigen, retinal neoplasm

## Abstract

Focal reactive nodular gliosis (FRNG) is an extremely rare benign retinal reactive astrocytic tumor that results from the proliferation of well-differentiated glial cells secondary to a variety of retinal conditions. We describe a case of this tumor in a 64-year-old male in association with a chorioretinal scar he has had since childhood. The symptom was sudden painful vision loss. In the clinical examination, iris rubeosis, posterior synechiae, cataract, vitreous haze and a suspected fundus mass were showed. B-scan ultrasonography demonstrated a retinal mass consistent with choroidal melanoma. The magnetic resonance imaging (MRI) showed a well-circumscribed mass with T1 hyperintensity and T2 hypointensity. Enucleation was performed and histopathologic and immunohistochemical studies confirmed the diagnosis of FRNG.

## Introduction

Gliosis refers to the activation and proliferation of glial cells in response to stimuli that disrupt neuroglial function, structure, and homeostasis. Müller cells and fibrous astrocytes constitute the 2 major types of glial cells [1]. Focal reactive nodular gliosis (FRNG) (retinal reactive astrocytic tumor) is an extremely rare, benign, intraocular tumor that results from the proliferation of well-differentiated glial cells secondary to a variety of degenerative, inflammatory, or ischemic retinal insults such as congenital abnormalities and malformations, trauma, vascular disorders, and retinal detachment, among others [2]. We report a rare case of FRNG in a patient with chronic blind eye, diagnosed from his enucleated globe through histopathologic and immunohistochemical studies.

## Case description

A 64-year-old male patient with sudden painful vision loss in the left eye (LE) was referred to our department for a detailed examination. He had an ophthalmological history of low vision in both eyes due to hypermetropic amblyopia in the right eye (RE) and a chorioretinal scar that he has had since childhood in the LE.

**Figure 1:**
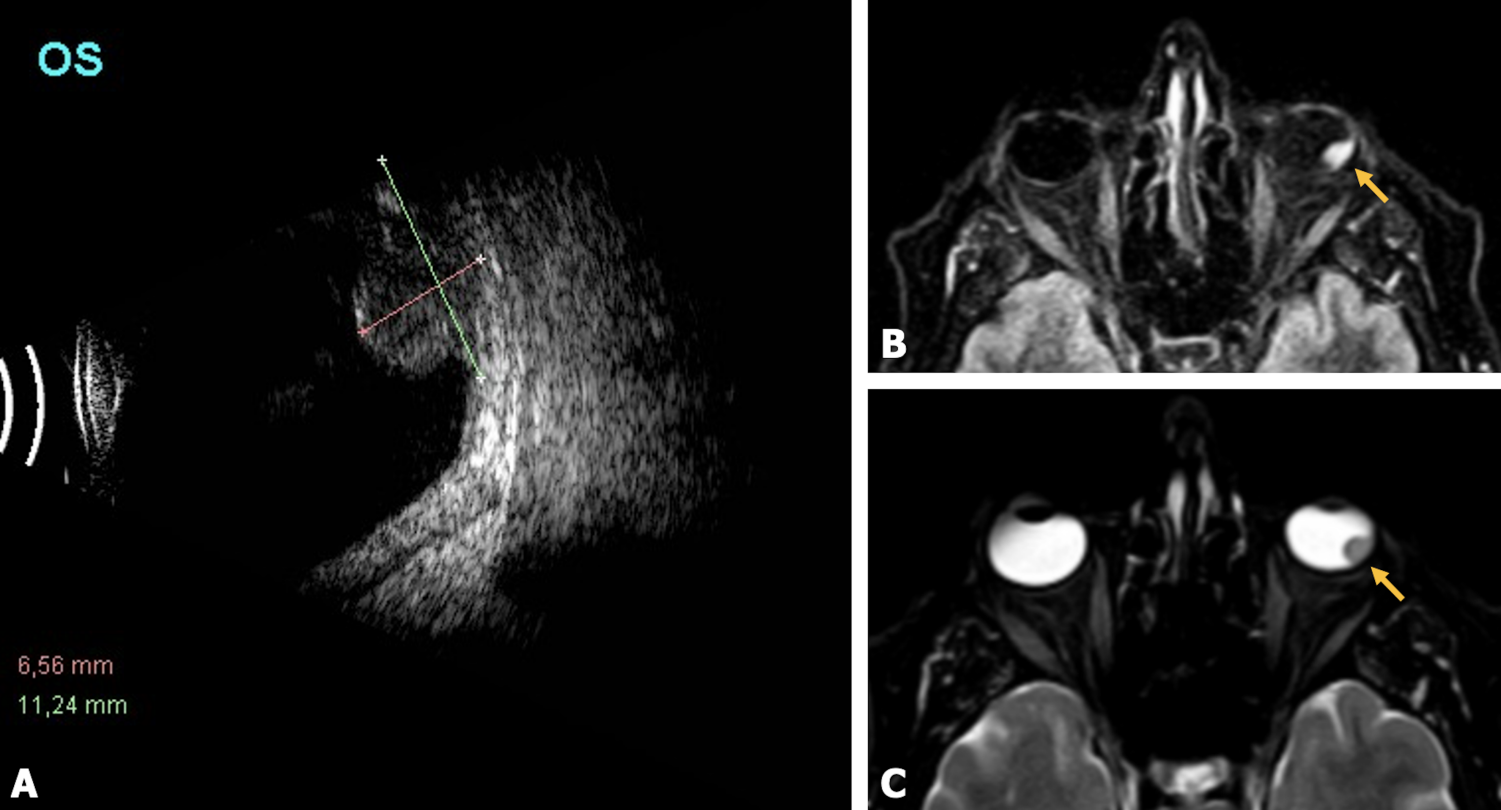
A: B-scan ultrasonography showing a mushroom-shaped mass protruding through the retina into the vitreous cavity, measuring 11.24 mm in the largest basal diameter and 6.56 mm in height. B: Sequence T1 and C: sequence T2 magnetic resonance imaging (MRI) showing a hyperintense and hypointense intraocular mass (yellow arrowhead), respectively, consistent with choroidal melanoma.

The recorded best-corrected visual acuity was 0.16 in the RE and light perception in the LE. An applanation tonometry revealed an intraocular pressure (IOP) of 38 mmHg in the LE. Anterior slit-lamp examination showed iris rubeosis, posterior synechiae and cataract. Fundus eye examination exhibited vitreous haze and hemorrhage in reabsorption with suspected inferior mass. In the RE IOP was within a normal range and the anterior segment and fundus examinations were unremarkable. B-scan ultraso-nography demonstrated an infero-temporal retinal mass, mushroom-shaped, measuring 11.24x8.2x6.56 mm (Figure 1A (Fig. 1)). Magnetic resonance imaging (MRI) was performed. It showed a well-circumscribed mass with hyperintensity in sequence T1 and hypointensity in sequence T2, consistent with choroidal melanoma (Figure 1B and C (Fig. 1)). Systemic extension test was negative for metastasis. Enucleation was performed on the basis of intraocular tumor consistent with choroidal melanoma with secondary vitreous hemorrhage and inflammatory signs.

**Figure 2:**
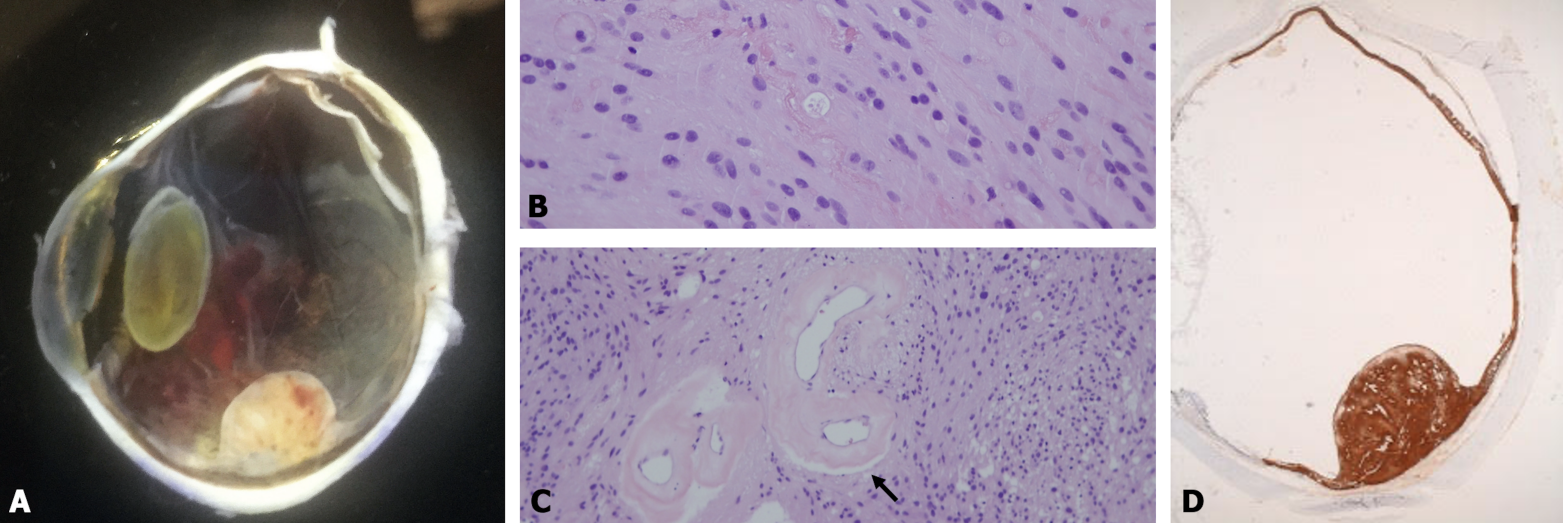
A: Enucleated eye with a nodular amelanotic retinal mass with respect of underlying choroid and projecting into the vitreous cavity. B: A histopathologic image of the focal reactive nodular gliosis demonstrates that the tumor consists of a mass of spindle-shaped eosinophilic astrocytes (hematoxylin and eosin, 400 X). C: Note the abnormal sclerotic blood vessels with thickened and hyalinized wall (black arrow) (hematoxylin and eosin, 100 X). D: Immunohistochemistry for GFAP. Glial fibrillary acid protein is strongly expressed in the components of the spindle cells (glial tissue marker).

Gross ocular pathology examination revealed an eye measuring 24x24x23 mm with no signs of extraocular extension. A nodular amelanotic mass was observed involving less than one quadrant of retina at equator with respect of underlying choroid and blurred overlying vitreous (Figure 2A (Fig. 2)). Histopathological examinations showed in hematoxylin and eosin (H&E) staining a well delimited nodular retinal proliferation composed of fusiform cells, arranged in bundles with hair-like processes with round or oval nucleus without signs of atypia. Numerous blood vessels with thickened and hyalinized wall were present as were hemorrhage foci (Figure 2B (Fig. 2)). Adjacent retina shows degeneration and intraretinal exudates. Immunohistochemical stains showed intense positivity for glial fibrillary acid protein (GFAP) (Figure 2C (Fig. 2)) but not for S-100 protein. Ki67 protein revealed <5% of proliferative activity. These findings were consistent with FRNG.

## Discussion

We describe a case of FRNG in association with chorioretinal scar since childhood, mimicking choroidal melanoma. It affects children more than adults, often occurs ≥10 years after the predisposing insult, and all ages and both sexes are affected with almost equal frequency [3]. Association between proliferation of retinal glial cells and chronic blind eyes is frequent [2].

Clinically it is difficult to distinguish from other intraocular neoplasms, and the differential diagnosis includes choroidal melanoma, retinal hemangioblastoma, astrocytic hamartoma, tumors of the retinal pigment epithelium, intraocular metastasis and vasoproliferative tumor of the retina (VPTR) [2].

The definitive diagnosis is secured by histopathologic and immunohistochemical features. The histopathologic hallmark of FRNG is the robust and apparently unmodulated proliferation of spindled and oval-shaped cells with fibrillated eosinophilic cytoplasm manifesting indistinct borders and banal nuclei [3]. FRNG and VPTR share similar histopathological features; while vascular components are largely represented in VPTR, FRNG is characterized by a glial/astrocytic-predominant proliferation [4]. The glial component was prominent in the present case. Strong and diffuse positive staining for GFAP aids in confirming the glial cell composition of the mass. GFAP is an intermediate filament protein significantly upregulated after retinal injury in both Müller and astrocytes cells. Müller cells extend through the full thickness of the retina from the podocytes beneath the internal limiting membrane to the external limiting membrane, and serve as sustentacular cells that maintain an equable environment for the retinal neurocytes. On proliferation, these cells can acquire a full complement of cytoplasmic glial filaments [5]. Astrocytes reinforce the outer layers of the retinal vessels to preserve the bloodretinal barrier of the inner retina, and have the capacity to synthesize vascular endothelial growth factor, thus engendering new vessel growth [1]. The low proliferation index with anti-Ki67 antibody immunostaining, as shown in this case, suggested the tumor was not malignant. Increasing Ki-67 positivity correlates with the progressive acquisition of aggressiveness in most tumors [6].

## Conclusion

To conclude, FRNG is a rare intraocular tumor resulting from the benign proliferation of well-differentiated glial cells. The clinical and ultrasonographic appearance may mimic choroidal malignant melanoma. We must consider it in association with a history of chronic blind eye. Histopathological examination and immunohistochemical markers are essential tools for an accurate diagnosis.

## Notes

### Competing interests

The authors declare that they have no competing interests.
